# Recognition of *Plasmodium falciparum* mature gametocyte-infected erythrocytes by antibodies of semi-immune adults and malaria-exposed children from Gabon

**DOI:** 10.1186/s12936-017-1827-7

**Published:** 2017-04-26

**Authors:** Tamirat Gebru, Anthony Ajua, Michael Theisen, Meral Esen, Ulysse Ateba Ngoa, Saadou Issifou, Ayola A. Adegnika, Peter G. Kremsner, Benjamin Mordmüller, Jana Held

**Affiliations:** 1 Institute of Tropical Medicine, University of Tübingen, Wilhelmstraße 27, 72074 Tübingen, Germany; 2 German Centre for Infection Research (DZIF), Partner Site Tübingen, Germany; 3 Centre de Recherches Médicales de Lambaréné (CERMEL), Lambaréné, Gabon; 4 German Centre for Infection Research (DZIF), Partner Site Lambaréné, Gabon; 5 Department of Medical Laboratory Sciences, College of Medical and Health Sciences, Haramaya University, Harar, Ethiopia; 6 Department for Congenital Disorders, Statens Serum Institut, Copenhagen, Denmark; 7 Center for Medical Parasitology at Department of International Health, Immunology and Microbiology, University of Copenhagen, Copenhagen, Denmark; 8 Department of Parasitology, Leiden University Medical Center, Leiden, The Netherlands; 9 Fondation pour la Recherche Scientifique (FORS), Cotonou, Benin

**Keywords:** Malaria, Transmission blocking, *Plasmodium falciparum*, Clinical isolates, Helminths, *Ascaris lumbricoides*, *Trichuris trichiura*, GMZ2, Immune modulation

## Abstract

**Background:**

Transmission of malaria from man to mosquito depends on the presence of gametocytes, the sexual stage of *Plasmodium* parasites in the infected host. Naturally acquired antibodies against gametocytes exist and may play a role in controlling transmission by limiting the gametocyte development in the circulation or by interrupting gamete development and fertilization in the mosquito following ingestion. So far, most studies on antibody responses to sexual stage antigens have focused on a subset of gametocyte-surface antigens, even though inhibitory Ab responses to other gametocyte antigens might also play a role in controlling gametocyte density and fertility. Limited information is available on natural antibody response to the surfaces of gametocyte-infected erythrocytes.

**Methods:**

Ab responses to surface antigens of erythrocytes infected by in vitro differentiated *Plasmodium falciparum* mature gametocytes were investigated in sera of semi-immune adults and malaria-exposed children. In addition, the effect of immunization with GMZ2, a blood stage malaria vaccine candidate, and the effect of intestinal helminth infection on the development of immunity to gametocytes of P. falciparum was evaluated in malaria-exposed children and adults from Gabon. Serum samples from two Phase I clinical trials conducted in Gabon were analysed by microscopic and flow-cytometric immunofluorescence assay.

**Results:**

Adults had a higher Ab response compared to children. Ab reactivity was significantly higher after fixation and permeabilization of parasitized erythrocytes. Following vaccination with the malaria vaccine candidate GMZ2, anti-gametocyte Ab concentration decreased in adults compared to baseline. Ab response to whole asexual stage antigens had a significant but weak positive correlation to anti-gametocyte Ab responses in adults, but not in children. Children infected with *Ascaris lumbricoides* had a significantly higher anti-gametocyte Ab response compared to non-infected children.

**Conclusion:**

The current data suggest that antigens exposed on the gametocyte-infected red blood cells are recognized by serum antibodies from malaria-exposed children and semi-immune adults. This anti-gametocyte immune response may be influenced by natural exposure and vaccination. Modulation of the natural immune response to gametocytes by co-infecting parasites should be investigated further and may have an important impact on malaria control strategies.

## Background

Malaria remains a major global public health problem affecting hundreds of millions of people annually, mainly in sub-Saharan Africa. Each year approximately half a million people die, mostly children younger than 5 years [1]. Gametocytes, the sexual stage of the parasites, are essential for transmission of the parasite from man to mosquito. Malaria transmission can be interrupted by drug treatment affecting gametocytes [2, 3], causal chemoprophylaxis, vector control as well as the acquisition of immunity to sexual stage parasites by the human host [4, 5].

Transmission blocking interventions that target gametocyte development and gamete fertilization are considered an essential part of malaria control, especially if containment or eradication of the disease is the aim. Transmission blocking vaccines (TBVs) would have a great public benefit in malaria-endemic countries by breaking the life cycle and decreasing the number of new infections. In addition, it is assumed that TBVs could help in containing the spread of parasites resistant to drugs or malaria vaccine components directed against asexual blood stage or pre-erythrocytic stages [6, 7]. To better understand immunity against the sexual stage of the plasmodial life cycle and for the design and development of TBVs, profiling the response to mature gametocytes is of relevance.

Antibodies (Abs) are important mediators of sexual stage immunity against *Plasmodium* and other apicomplexan parasites [8–12]. Such Abs can affect malaria transmission either by inhibiting gametocyte development [5] or by directly affecting viability of mature sexual stages [13–15]. The latter might happen within the body or once they are ingested by mosquitoes [5, 16–18], e.g. through opsonization of gametes followed by phagocytosis [12]. In malaria-endemic areas, the age-dependent decline of the duration of gametocyte carriage [19, 20] is most likely due to an increase in gametocyte exposure and development of sexual stage specific immune responses, in parallel to the asexual immunity acquired with age [21].

Indirectly, immune responses to asexual stage antigens may decrease transmission by limiting the number of asexual parasites that develop to gametocytes [21], similar to the decrease of gametocytogenesis that results from the elimination of asexual infections by drugs [22]. However, development of sexual-stage immunity is different from the immune response directed to asexual stage antigens [13, 15]. Gametocytes have distinct gene expression patterns [23] and proteomic profiles [24] compared to asexual stages. Similarly early and late stage gametocytes differ; for example, the latter have a comparatively low representation of active export machinery proteins. However, some overlaps are expected in the proteomic profiles and exported proteins between the different stages of the parasite’s life cycle [24].

Naturally acquired sexual-stage antibodies are produced against gametocyte-infected erythrocyte surface antigens or gamete-specific antigens in the circulation and also against mosquito-stage parasites that act following ingestion of the parasite [25]. There are only few studies on natural immune responses to gametocyte-infected erythrocyte surface antigens. Saeed et al. [15] showed that 34% of Gambian children had plasma antibodies recognizing stage V gametocyte-infected erythrocytes in vitro, with no recognition of stages I–IV. In the same study Abs to gametocyte surface antigens were associated with lower gametocyte densities indicating the importance of Abs in reducing gametocyte carriage. Most other studies on immune responses to sexual stage antigens have focused on few specific antigens, mainly the TBV candidates Pfs230 [18, 26–31] and Pfs48/45 [18, 27–32]. The association of Ab response to these single antigens and transmission reducing activity is not consistent. After testing antibody response to both antigens, some authors reported a correlation of transmission reduction with both antigens [31], while others found associations only with Pfs230 [18, 28] or only with Pfs48/45 [29, 30]. Even though correlation might be confounded by exposure history to earlier malaria infections, these results suggest that Ab responses to other gametocyte-specific antigens may play an additional role in controlling transmission [5]. Here, Ab responses to gametocyte-infected erythrocyte surface antigens were measured in individuals from a malaria-endemic country (Gabon).

In the present study, the concentration of anti-gametocyte Abs against in vitro differentiated mature gametocytes of one *Plasmodium falciparum* clinical isolate and one laboratory strain (NF54) was measured by flow cytometric immunofluorescence assay (IFA) in sera from malaria-exposed children and semi-immune adults. Since exposure to asexual blood stage antigens and co-infection with other highly prevalent parasites may modulate immune responses [33, 34], here the anti-gametocyte responses were related to infection status with intestinal helminths. Assuming a reduced anti-gametocyte antibody response after vaccination with a malarial vaccine, additionally the anti-gametocyte antibody response to antibodies induced by vaccination was related with the asexual blood stage vaccine candidate GMZ2, a recombinant fusion protein of *P. falciparum* glutamate-rich protein (GLURP) and merozoite surface protein 3 (MSP3) [35]. Therefore, the collected serum samples during the GMZ2 trials were used to investigate this in depth.

## Methods

### Study samples and sources

Gametocyte recognition of antibodies were investigated in adults and children who had a different history of exposures to malaria and infection status with intestinal helminths. The serum samples analysed in the current investigation are from two studies [36, 37] conducted between July 2007 and October 2008 at the Centre de Recherches Médicales de Lambaréné (CERMEL), Lambaréné in Gabon. The two sets of serum samples were from Phase I clinical trials designed to assess safety and immunogenicity of GMZ2, an experimental blood-stage malaria vaccine candidate, in malaria-exposed adults and children, respectively [36, 37], which shows partial efficacy [38].

As part of the GMZ2 trials, samples were collected from 30 children aged 1–5 years [36] and 40 semi-immune adults (18–45 years) [37]. At the time of this investigation samples were only available for 36 adults. Samples collected before (Day 0) and on Day 84 after vaccination (4 weeks after the last immunization) were analysed to assess if vaccination has an effect on the development of antibody response to the sexual stages of *P. falciparum*. During the GMZ2 trials, participants received three doses of either 30 or 100 μg GMZ2, adjuvanted with aluminum hydroxide or a control vaccine (Verorab) and were followed for 1 year. Further details of the studies are published elsewhere [36, 37]. Serum samples were stored at −80 °C until analysed.

The studies were conducted according to the principles of the Declaration of Helsinki and good clinical practice guidelines. The respective competent ethics committees: the Comité d’Ethique Régional Indépendant de Lambaréné, CERIL and the Gabonese Ministry of Health reviewed and approved the studies. A signed informed consent was obtained from each participant or parent/guardian of participant aged less than 18 years. Analysis of Ab levels against *P. falciparum* was part of the respective study protocols.

### Gametocyte culture and purification

Gametocyte culture was initiated from a continuous culture of asexual *P. falciparum* parasites as described earlier [39]. Briefly, the asexual cultures were kept at 5% haematocrit and less than 2% parasitaemia at 37 °C, 5% CO_2_ and 5% O_2_ with weekly sorbitol synchronization and daily change of medium (RPMI 1640 supplemented with 25 mM HEPES, 28 mM NaHCO_3_, 50 μg/mL gentamycin, 0.5% w/v Albumax II, 2.4 mM l-glutamine, and 0.14 mM hypoxanthine); medium of the clinical isolate contained in addition 5% human serum. The *P. falciparum* laboratory strain NF54 (Sanaria Inc., Rockville, MD, USA) and a laboratory adapted clinical isolate from Lambaréné, Gabon where the samples had been collected were used for the experiments. The clinical isolate (JH013) was cultivated from a blood sample collected in 2009 from an individual with *P. falciparum* mono-infection and cryopreserved at −150 °C in glycerolyte as reported previously [40].

Gametocyte culture was performed as described earlier [41, 42] with some modifications. Human serum (5%) was added to the gametocyte growth media and the culture was initiated with a parasitaemia of 0.5 and 9% haematocrit and kept at 5% O_2_/CO_2_ at 37 °C with daily change of medium without parasite dilution. On Day 7 the haematocrit was lowered to 4.5% by doubling the volume of medium. Beginning with Day 11 the parasites were treated with 50 mM *N*-acetyl-d-glucosamine (MP Biomedicals GmbH, Santa Ana, CA, USA) for 4 days to eliminate asexual stage parasites.

To enrich stage V gametocytes from approximately 2% to more than 90% on Day 15 a density gradient centrifugation (800*g* for 20 min) on 33% NycoPrep 1.077 cushions (AXIS-SHIELD PoC AS, Oslo, Norway) followed by magnetic separation with LD-MACS magnetic columns (Miltenyi Biotec, Gladbach, Germany) was performed.

### Fluorescence microscopy and flow cytometry-based IFA

For IFA, gametocyte-infected erythrocytes were analyzed in three different ways: live [15], fixed, or fixed and permeabilized [43]. For assessment of Ab response to plasmodial antigens, a *P. falciparum* culture was used, that was enriched for late developmental stages of asexual [44] and stage V gametocytes. In brief, for fixation, parasites were incubated for 30 min in a mixture of 4% Electron Microscopy (EM) grade paraformaldehyde (Merck, Germany) and 0.0075% EM grade glutaraldehyde (Sigma-Aldrich, Germany), then washed once with PBS and stored at 4 °C until the IFA was performed. Prior to Ab staining, a fraction of fixed cells was permeabilized by PBS/0.1% Triton-X-100 for 10 min and washed once with PBS.

All live, fixed or fixed and permeabilized parasites were blocked using PBS/3%BSA for 1 h before the addition of test or negative control sera. Following the blocking step, evaluation of anti-gametocyte Ab response was done using cytometry-based IFA as described earlier [44]. Serum samples (in PBS/3% BSA diluted 1/4000 and 1/50, respectively) from adults and children were added and incubated for 1 h. Serum dilutions that gave the best discrimination between negative and positive were used. In the cohort children are less reactive compared to adults, hence lower dilutions were used [45, 46]. After three washes with PBS, secondary Ab staining was carried out for 1 h with Alexa Fluor 488-labeled rabbit anti-human IgG (Life Technologies GmbH, Darmstadt, Germany) at a 1:3000 dilution and washed three times before the quality of the staining was assessed by fluorescence microscopy.

Subsequent acquisition of flow cytometry data was done using a FACSCanto II cytometer equipped with the FACSDiva software version 6.1.2 (BD Biosciences, San Jose, USA). Fluorescence of each event was analysed and the result expressed as percentage of positive fluorescent cells (PPFC) based on 20,000 erythrocytes (events) acquired.

### ELISA for measurement of Abs to GMZ2 antigens

For comparison, a subset of previously reported GMZ2-specific IgG ELISA values (Days 0 and 84) of clinical trial participants were used for the current analyses [36, 37]. ELISA data were generated using a standardized ELISA protocol as described previously [36, 37].

### Stool examination for worm infection

During follow up of study participants enrolled in the Phase I paediatric clinical trial of GMZ2 [36], stool samples were collected. They were freshly examined to evaluate the prevalence of soil-transmitted helminths at enrolment and on Day 84 as reported earlier [34].

### Statistical analysis

GraphPad Prism version 5.0 (GraphPad Software, San Diego California) and SPSS version 16 were used for statistical analysis. Nonparametric tests were used for statistical testing. The significance level was set to a two-sided alpha of 0.05 and corrected using the Bonferroni method where applicable. Paired samples were tested with the Wilcoxon signed-rank test whereas the Mann–Whitney test was applied in case of unpaired data. Spearman correlation between Ab responses to whole sexual and asexual stage (including recombinant GMZ2) antigens were calculated and presented with 95% confidence intervals [47]. A sample was declared sero-negative by cytometric IFA if the percentage gated cells positive for a given marker was within three standard deviations above the mean of the negative controls (serum samples from malaria naïve individuals).

## Results

### Recognition of gametocyte antigens by Abs is enhanced by fixation and permeabilization

**Fig. 1:**
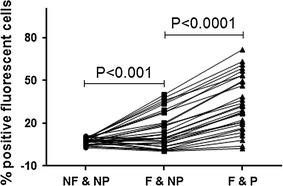
Antibody response to in vitro differentiated live, fixed or permeabilized *Plasmodium falciparum* gametocytes. The figure shows the flow cytometric data obtained using live, fixed or permeabilized gametocyte-infected erythrocytes. *Dots* represent percentage positive cells (erythrocytes positive for AlexaFluor-488), one dot per tested sample. Gates were set against controls, which are serum from malaria naïve individuals. *NF* non fixed, *NP* non permeabilized, *F* fixed, *P* permeabilized. The *Y-axis* shows percentage of erythrocytes positive for the used marker (Alexa.Fluor-488)

**Fig. 2:**
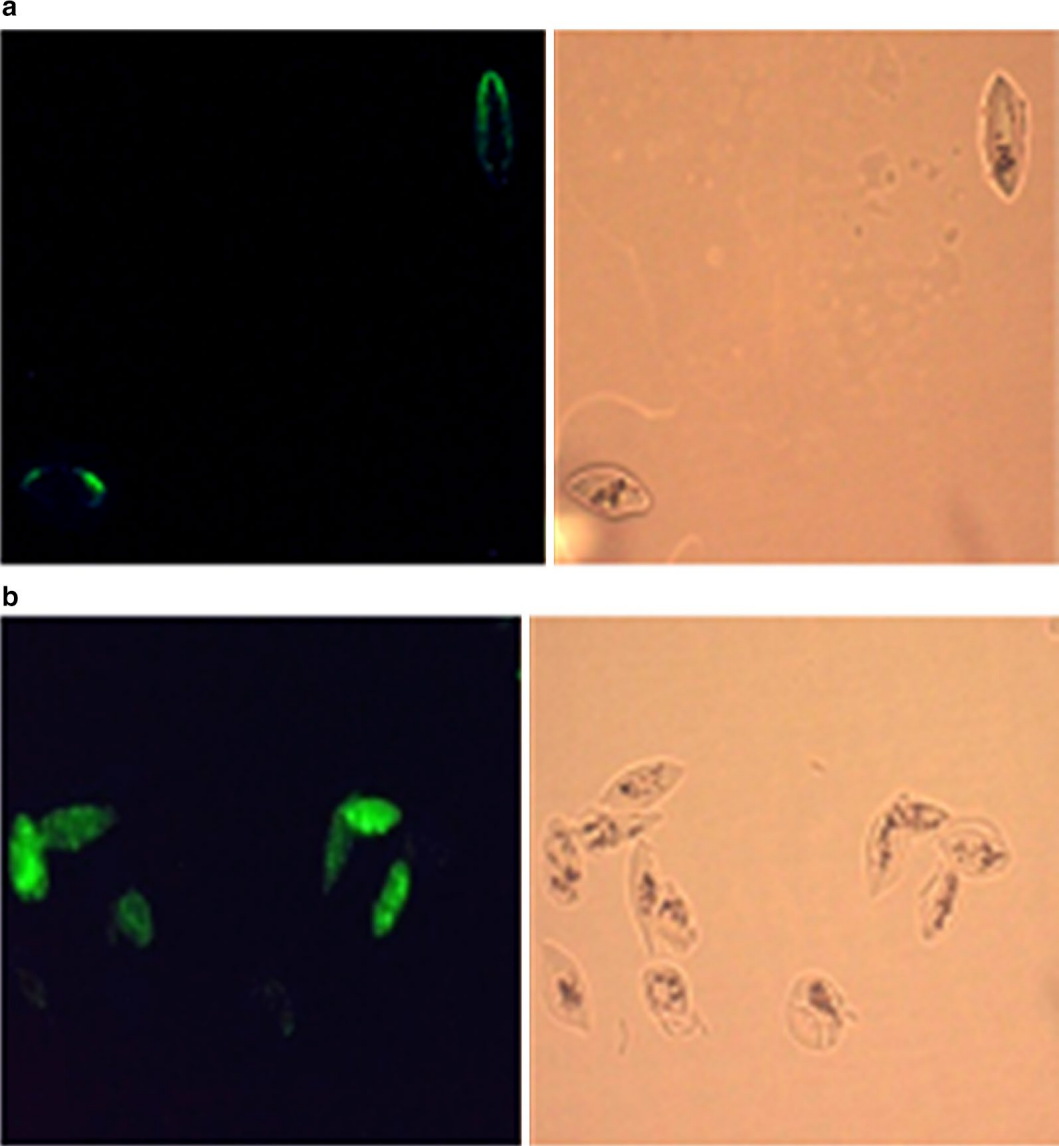
Microscopic IFA of antibody response to *P. falciparum* gametocytes. The figures show an example picture of AlexaFluor-488 stained parasites by fluorescence microscopy before (**a**) and after (**b**) permeabilization

The level and prevalence of Ab response to *P. falciparum* mature gametocytes was evaluated in sera from adults (n = 36) and children (n = 30) from the two GMZ2 trials (Table 1) using live, fixed or fixed and permeabilized NF54 *P. falciparum* mature gametocyte infected erythrocytes. As expected, the level of antigen recognition by Abs was significantly higher after fixation and permeabilization of NF54 *P. falciparum* gametocyte-infected erythrocytes, two-fold and four-fold change, respectively (Fig. 1). Apparently, immune recognition of the parasite includes antigens not expressed on the surface of erythrocytes (Fig. 2a, b). However, these intracellular antigens could also be shared by the asexual and sexual antigens of the parasite. Therefore, for all subsequent measurements, non-fixed and non-permeabilized gametocyte-infected erythrocytes were used.

**Table 1:** Study name, background characteristics, grouping, hematological profiles and time of data collection of samples used within the study

Study identifier (population)	Sampling period (month/year)	No. of participants	Median age in years (range)	Mean hemoglobin (g/dL)	Vaccine allocation	Samples time points
GMZ2_3_08 *(Children)* [36]	09/2008–10/2009	30	3.5 (1–5)	10.5 10.5 10.3	GMZ2 100 µg GMZ2 30 µg Rabies	Days 0 and 84
GMZ2_2_07 *(Adults)* [37]	07/2007–08/2008	32	26.3 (18–45)	13.8 14.1	GMZ2 100 µg Rabies	Days 0 and 84

### Anti-gametocyte Ab responses of malaria-exposed individuals in different *P. falciparum* parasite strains

**Fig. 3:**
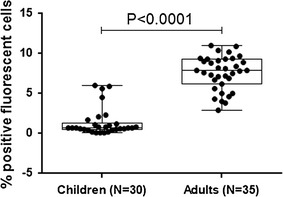
Baseline levels of antibody response to gametocytes of *P. falciparum* in semi-immune adults and malaria exposed children. Antibody response (percentage of positive cells) against in vitro developed matured gametocytes (*P. falciparum* NF54 strain) of the serum samples of the different trials conducted in Gabon (GMZ2 children on Day 0 [36] and GMZ2 adults on Day 0 [37]) at baseline. Median, 25th and 75th percentiles, and minimum and maximum ranges are indicated

The anti-gametocyte Ab-binding patterns of one clinical isolate (JH013) and NF54 were compared to identify the strain that is best recognized by participants’ Abs [37]. There was no significant difference between the two strains in the recognition of gametocytes by serum antibodies from adults and children of the studies and, therefore, further experiments were done only with the *P. falciparum* laboratory strain NF54. The Ab response to mature gametocytes was higher in adults compared to children (Fig. 3), as well as the seroprevalence of anti-gametocyte Ab response (77% in adults and 57% in children).

### Asexual and sexual stage anti-malarial immune response in GMZ2 vaccinated adults and children

**Fig. 4:**
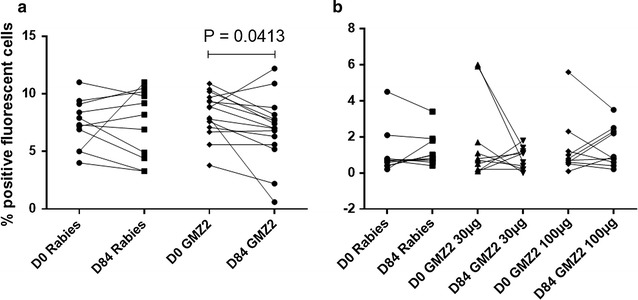
Effect of GMZ2 vaccination on the development of antibody response to mature *P. falciparum* gametocytes. **a**, **b** Show the antibody response in semi-immune adults and malaria-exposed children, respectively. In adults, 73.3% (11/15) show a decrease of Ab response after vaccination while 20% (3/15) show an increase and one person has shown no change (6.7%). *D0* Day 0, *D84* Day 84, GMZ2 100 µg or GMZ2 30 µg: participants who received 100 or 30 µg of GMZ2 vaccine, respectively. Rabies: participants who received the control rabies vaccine

The effect of GMZ2 vaccination on the development of anti-gametocyte immune response in adults and children was assessed. It was hypothesized that immune responses generated by the GMZ2 vaccine may divert the immune response towards the vaccine or lead to a lower gametocyte density, despite its relatively low efficacy (11–14%) in children between 1 and 5 years [38]. Interestingly, the anti-gametocyte Ab response in GMZ2 vaccinated adult individuals was lower on Day 84 after vaccination compared to baseline (Day 0) (Fig. 4a). In children, the anti-gametocyte Ab response was not different after vaccination in both, the GMZ2 and rabies vaccinated control group (Fig. 4b).

Since sexual stage immunity may be developed independently from asexual-stage immunity [13, 15], possible associations were tested between sexual stage immune response (anti-gametocyte Abs) to the asexual stage immunity (Abs against asexual blood stages) and Ab responses to the GMZ2 vaccine antigen in the two vaccinated groups. For this analysis, our previously published data on anti-plasmodial (against whole asexual stage antigens) [44] and anti-GMZ2 [36, 37] Abs were used to assess correlations with anti-gametocyte Abs. There was a positive correlation on Day 0 before vaccination between the sexual and asexual Ab responses in the semi-immune population (Table 2). No correlation was found in data from children. The Ab response to GMZ2 was not associated with sexual Ab response neither in adults nor in children (Table 2).

**Table 2:** Spearman correlation of the antibody response to sexual stage antigens with asexual stage antigens and the GMZ2 vaccine antigen of *P. falciparum* in adults/children from Gabon

Study participants and vaccination	Antibody against whole asexual stage antigens by C-IFA (Mean PPFC)	Antibody against GMZ2 by ELISA (Mean Ab titers)
	Rho (95% CI)	Rho (95% CI)
Antibody against stage V gametocytes by C-IFA (Mean PPFC)		
Adults		
D0^a^	0.39 (0.03, 0.66)*	0.17 (−0.18, 0.48)
D84 (GMZ2)	0.41 (−0.17, 0.78)	0.44 (−0.13, 0.80)
D84 (Rabies)	0.40 (−0.21, 0.79)	0.54 (−0.03, 0.85)
Children		
D0^a^	0.16 (−0.24, 0.51)	0.28 (−0.11, 0.60)
D84 (GMZ2, 30 µg dose)	0.02 (−0.62, 0.64)	−0.10 (−0.69, 0.57)
D84 (GMZ2, 100 µg dose)	0.06 (−0.59, 0.67)	0.22 (−0.48, 0.75)
D84 (Rabies)	−0.27 (−0.77, 0.44)	0.22 (−0.48, 0.75)

*C*-*IFA* Cytometric immunofluorescence assay, *ELISA* Enzyme-linked immunosorbent assay, *PPFC* percent positive fluorescent cells, *CI* Confidence interval

Significant p value is indicated as follows: * P < 0.05

^a^ *D0* Values represent both GMZ2 and Rabies groups together. *D0* Day 0, *D84* Day 84

### Effect of helminth infection on development of anti-gametocyte immunity

**Fig. 5:**
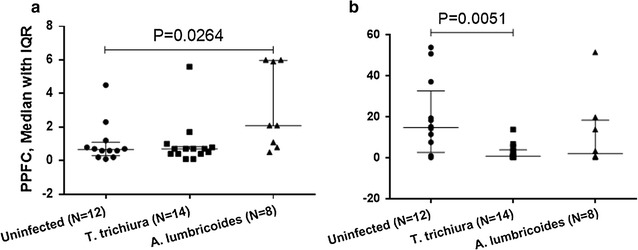
Antibody response to *P. falciparum* mature gametocytes in children from Gabon infected with intestinal parasites. The *x*-*axis* shows helminth infection status on Day 0 before vaccination in children, N = 30. **a**, **b** show antibody recognition of live and permeabilized gametocyte infected erythrocytes, respectively. *Y*-*axis* shows percentage of erythrocytes positive for AlexaFluor-488. *PPFC*  % positive fluorescent cells, *IQR* Interquartile range. Median and IQR are indicated

The anti-plasmodial immune response is modulated by co-infection of *P. falciparum* with other infectious agents including helminths [48–50]. Previously an effect of intestinal helminths on anti-GMZ2 responses in 20 GMZ2 vaccinated children was found [34] and, therefore, also in this study the effect of intestinal parasites co-infection on the modulation of anti-gametocyte immune responses in children was assessed. Parasitological data of all 30 children included in the GMZ2 Phase Ib trial [36] were used to explore the modulation of anti-gametocyte immune response by helminths. Overall, five different helminths were recorded. *Trichuris trichiura* and *Ascaris lumbricoides* were present in a relative high proportion on enrolment (14/30; 47% and 8/30; 27%) and on Day 84 (15/30; 50% and 11/30; 37%), respectively. There was also a low rate of infections (<7%) by *Strongyloides*, *Schistosoma*, and *Ancylostoma*. Interestingly, children infected with *Ascaris lumbricoides* had significantly higher anti-gametocyte Ab response compared to uninfected children when using live non-permeabilized gametocytes (Fig. 5a). When the assay was done using fixed and permeabilized parasites, a significantly lower Ab response was observed in *Trichuris trichiura*-infected children (Fig. 5b).

## Discussion

Understanding the development of Ab responses to sexual stages of *P. falciparum* in malaria-exposed populations is important for understanding transmission patterns and the design and development of TBVs. In this study in a highly malaria endemic area, adults showed higher Ab responses to sexual stage antigens than children. The effect of age on anti-gametocyte Ab production and transmission reducing activity has been addressed in several studies and resulted in contradictory results. Some have shown an increase in antibody response to fixed whole parasites [51] and to Pfs48/45 and Pfs230 with age [46], others a decline in transmission reducing activity of sera with age [27]. The difference may be due to the varying age ranges, different epidemiological settings and inconsistent assays for anti-gametocyte Ab measurements between the studies. Since effective humoral immunity to intraerythrocytic gametocytes requires prolonged exposure to the parasite [52], the epidemiological setting shall play an important role and the interpretation of divergent results needs to account for that.

The anti-gametocyte seropositivity rate was 77% in adults and 57% in children. Similar seropositivity rates have been recorded earlier, more than 50% in Ghanaian children with asymptomatic infection [53] and 34% in Gambian children with uncomplicated malaria and gametocytaemia [15]. In another cohort of Gambian children, who were gametocytaemic, an Ab response was detected in all participants [51]. This response might have been so high, as they fixed gametocytes with acetone that simultaneously permeabilizes the cell [54]. The same study reported a lower (42%) sero-prevalence when live gametes were used. Unfortunately, gametocyte carriage rates were not recorded in our studies. Due to different methodologies, direct comparison to the previously reported results is not possible. Of note, gametocyte prevalence is not always associated to antibody response to gametocytes [55] and transmission capacity [56, 57].

The effect of anti-GMZ2 Ab and Ab responses to whole asexual stage antigens and the effect of GMZ2 vaccination on the development of sexual-stage immunity was evaluated. The result showed a significant but weak positive correlation between Ab response to gametocyte antigens and whole asexual stage antigens, but not to GMZ2 antigen in adults, showing that exposure is correlated to Ab response to gametocytes in the investigated population. The level of sexual Ab response was significantly reduced after vaccination in GMZ2 vaccinated adults, but this difference was not seen in the rabies control group. An effective asexual malaria vaccine should lead to a reduced asexual parasite load and, therefore, reduced gametocyte production. The recently reported results of the GMZ2 vaccine shows 11–14% efficacy in children [38], therefore, the expected effect on gametocytes would be rather low. There is no evidence for the expression of either of the GMZ2 component proteins (MSP3 and GLURP) on the surface of gametocyte infected erythrocytes even though it was previously reported that GLURP is expressed in different stages of the parasite life cycle including the pre-erythrocytic stage [58]. The results show no boosting of the anti-gametocyte Ab response, supporting the data that none of the components is present at the sexual stage.

In children, there was no correlation between the Ab response to gametocyte antigens and GMZ2 (and other asexual antigens). The difference of the correlation results between adults and children might be due to the very low level of anti-gametocyte immune response in children complicating to see differences between groups. Additionally, Ab response to matured gametocytes did not change in children following vaccination with GMZ2 or the rabies control vaccine. Unfortunately our analysis has been underpowered to detect subtle differences in the study populations. Our result shall be confirmed with a bigger sample size.

As expected, antigen recognition by immune sera was significantly enhanced after fixation and permeabilization of gametocyte-infected erythrocytes as reported earlier [51]. This effect may be partly due to intracellular targets of transmission-blocking Abs [52] that are not exposed on the infected erythrocyte surface. However, the response measured after fixation and permeabilization of cells might not be gametocyte specific, but represent a response to the cocktail of internal asexual and sexual stage proteins [59]. Therefore, non-fixed and non-permeabilized gametocyte-infected erythrocytes were used to analyse the effect of age, vaccination, coinfection, and parasite strain variation on Ab responses to gametocytes. The natural Ab response to gametocyte antigens might inhibit gametocyte development and thereby interrupt the transmission of the parasite as shown in vitro by co-cultivation of early gametocytes with plasma from malaria patients [5]. In addition, Abs may also act following exflagellation in the mosquito midgut [60]. To get a deeper insight, characterization of naturally acquired transmission blocking Abs might improve the portfolio of TBVs.

Recognition of *P. falciparum* gametocytes of the laboratory strain NF54 and a clinical isolate by serum antibodies of semi-immune adults was assessed. Both lab strain and clinical isolate have been detected by the serum antibodies and no significant difference in the level of Ab recognition of the two strains could be seen. This was unexpected as the clinical isolate was collected from the area where the study participants were recruited. However, similar to the finding presented here, it has been shown earlier by Dinko et al. that the plasma antibodies from Ghanian participants recognizing the laboratory strain 3D7 and a clinical isolate from Kenia [55].

The immune response to malaria is modulated by co-infection with other infectious agents [33]. There were lower Ab responses in *Trichuris trichiura* infected children when the assay was done with permeabilized gametocytes. However this might not represent the suppression of the immune response to gametocytes but rather the response to the asexual or both stages of the parasite. This corroborates earlier findings in the same population, which showed that *Trichuris trichiura* infection is associated with 3.4-fold reduced Ab levels to the blood-stage asexual GMZ2 vaccine antigen while the response was increased in *Ascaris lumbricoides*-infected participants [34]. However, the effect of *Trichuris trichiura* infection could not be seen in the assay using non-permeabilized gametocyte-infected erythrocytes. In line with this, a cross-sectional study has reported a significant reduction of gametocyte specific (Pfs48/45) antibody titers in individuals infected with *Schistosoma haematobium,* though this effect is not seen when comparing another gametocyte specific antigen (Pfs230) [61].

The response to any gamete-specific antigens was not measured and therefore activation of non-fixed gametocytes cannot be ruled out. Mature gametocytes should be handled with care and a drop in temperature could provoke exflagellation. Therefore, the small fraction of recognized erythrocytes with gametocytes could also be a small fraction that was accidentally activated. However, we tried to ensure that non-fixed and non-permeabilized erythrocytes in the assay were intact and controlled for exflagellation by microscopy. Therefore, the measured response most probably represents largely the antibody response to antigens displayed on the surface of gametocyte-infected erythrocytes. Another explanation for the low response could be that only a fraction of gametocytes transport proteins to the outside and express surface proteins on the surface of erythrocytes. A reduced transport of proteins to the erythrocyte surface is known for asexual parasites of laboratory strains in long-term culture. To check this, the prevalence of positive cells was tested also for one clinical isolate (JH013), but did not find a difference in the percentage of recognized cells when compared to NF54.

Infection with *Ascaris lumbricoides* resulted in an increased anti-gametocyte immune response compared to the uninfected participants. This was surprising but similar effects on the anti-malarial immune response have been observed earlier [34]. However, due to the relatively small sample size and exploratory nature of the experiment, confirmatory studies will be required. Therefore, monitoring of anti-gametocyte responses should be done in further larger studies. In addition, it would be interesting to validate the immuno-modulatory effect of co-infections on the development of asexual and sexual stage immune responses and the transmissibility of malaria in other co-endemic areas.

## Conclusions

The level of Ab responses to mature gametocytes can be measured using flow cytometry. Adults show higher Ab responses when compared to children. Anti-gametocyte Ab responses are enhanced following permeabilization but may include responses to asexual antigens. Helminth infections and anti-malarial interventions modulate the humoral immune response to asexual and sexual blood stage *P. falciparum* parasitaemia. This may have a significant impact on malaria control strategies with the aim to reduce transmission of malaria.

## Abbreviations

Abs: antibodies
*A. lumbricoides*: *Ascaris lumbricoides*
ELISA: enzyme linked immunosorbent assay
IFA: immunofluorescence assay
TBV: transmission blocking vaccines
*T. trichiura*: *Trichuris trichiura*
